# Developing centrifugal force real-time digital PCR for detecting extremely low DNA concentration

**DOI:** 10.1038/s41598-024-62199-5

**Published:** 2024-05-21

**Authors:** Jong Cheol Shin, Jeong-Yeon Jeong, Seon Gyu Son, Sang-Haeng Choi, Ho-Chul Nam, Tae-Ho Yoon, Hyo-Jun Kim, Dong-Geun Choi, Hwarang Lee, Ukyeol Lee, Seon-Mo Yang, Il Kang, Dae-Young Jung, Han Woo Lee, Moon-Keun Lee, Tae Jae Lee, Geehong Kim, Han-Oh Park, Sung-Woon Lee

**Affiliations:** 1 RevoSketch Inc., Daejeon, Republic of Korea; 2 Center for Nano Bio Development, National NanoFab Center (NNFC), Daejeon, Republic of Korea; 3 Nano-Convergence Systems Research Division, Korea Institute of Machinery & Materials, Daejeon, Republic of Korea; 4 Bioneer Corporation, Daejeon, Republic of Korea

**Keywords:** Molecular biology, Engineering

## Abstract

Digital PCR (dPCR) is a technique for absolute quantification of nucleic acid molecules. To develop a dPCR technique that enables more accurate nucleic acid detection and quantification, we established a novel dPCR apparatus known as centrifugal force real-time dPCR (crdPCR). This system is efficient than other systems with only 2.14% liquid loss by dispensing samples using centrifugal force. Moreover, we applied a technique for analyzing the real-time graph of the each micro-wells and distinguishing true/false positives using artificial intelligence to mitigate the rain, a persistent issue with dPCR. The limits of detection and quantification were 1.38 and 4.19 copies/μL, respectively, showing a two-fold higher sensitivity than that of other comparable devices. With the integration of this new technology, crdPCR will significantly contribute to research on next-generation PCR targeting absolute micro-analysis.

## Introduction

The emergence of infectious diseases, such as COVID-19, necessary the growing need for precise nucleic acid measurements. Advances in polymerase chain reaction (PCR) have enabled more accurate nucleic acid detection and quantification, with real-time quantitative PCR being the predominant standard^1^. However, recently a more advanced technology, digital PCR (dPCR), achieved more precise quantification^2–7^. Unlike conventional PCR, the sample in dPCR is separated into significantly more partitions, and the reaction is performed in each partition individually, enabling digital-level DNA amplification and quantification^8^. Subsequent developments in microfluidics, which compartmentalized DNA molecules into volumes ranging from nanoliters to picolitres in formats, such as microchambers or micro-wells, provided more efficient partitioning methods, further enhancing the accuracy of dPCR analysis^9,10^. dPCR has been applied in various areas, such as biomarker development^10^, food safety^11^, forensic research, cancer diagnosis and detection^12–14^, pathogen detection^15–17^, rare allele gene detection^18^, infectious diseases detection^19^, and sample preparation for next-generation sequencing^20^. It has been most extensively used for detecting low-copy samples^21,22^ and copy number variations^23,24^. Furthermore, dPCR is a high-precision end-point measurement technique obviating the need for a reference samples, and has recently gained traction as a substitute for determining the concentration of defined nucleic acids in solution^25–28^. Additionally, nucleic acid targets should be randomly distributed within the partitions, and PCR conditions should be optimized to amplify each copy of the target. Notably, the molecular distribution in the partitions follows a Poisson distribution, which provides the basis for quantification using dPCR^29^. Since calculating the concentration involves dividing the copy number estimate by the analyzed volume, knowing the precise partition and liquid volume is essential when measuring concentration using dPCR^27,30^. However, most dPCR devices are limited in accurately determining the liquid volume, and present the problem of the introduced solutions not being fully utilized^31,32^. Additionally, results based on end-point detection using dPCR face challenges in eliminating false positives. The division into positive and negative droplets is often not clear, as some droplets exhibit intermediate fluorescence values, appearing as “rain” in the plot. This ambiguity can lead to a positive judgment error of up to 17% depending on the application point of the threshold^33,34^. In this study, a novel dPCR device, the centrifugal force real-time digital PCR (crdPCR), was introduced to address these challenges and optimize space efficiency. The crdPCR is an all-in-one device that performs sample partitioning, thermal cycling, and reading within a simple dedicated disk. Furthermore, a novel analysis method was devised using artificial intelligence techniques for real-time dPCR implementation, and its utility as a diagnostic tool was evaluated by comparing its performance with the most extensively used QX200™ droplet digital PCR System (Bio-rad) and QIAcuity™ Digital PCR system (Qiagen)^35^.

## Results

### Novel digital PCR platform

**Figure 1:**
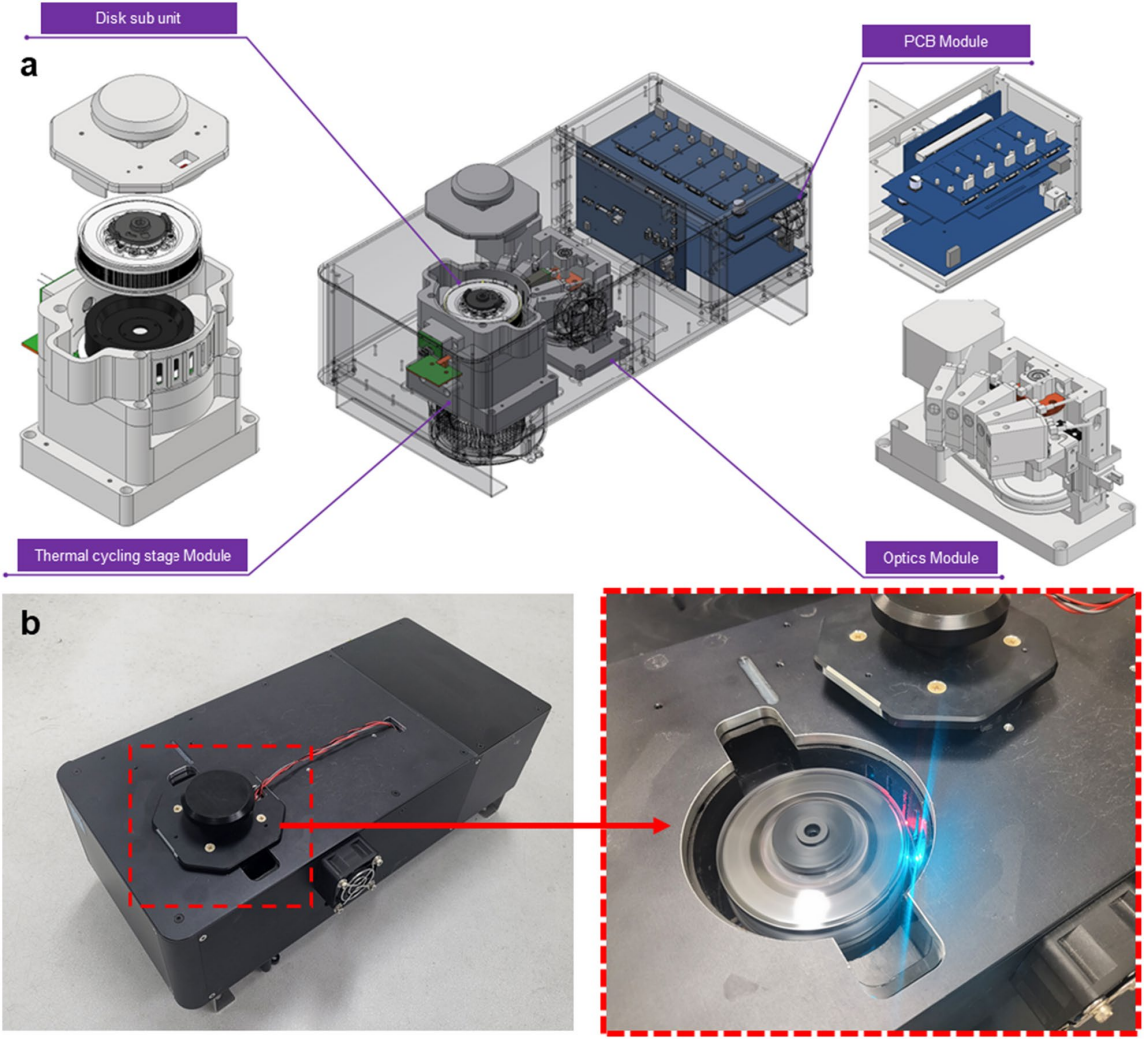
(**a**) Schematic of the digiQuark system with thermal control, fluorescent optical modules, and PCB modules. (**b**) Photographs of the implemented equipment and its behavior.

An all-in-one crdPCR platform, called digiQuark, that incorporates a rotating centrifugal force-based partitioning system featuring a dedicated disk with micro-wells was developed (Fig. 1). This platform has a compact size of 185 × 395 × 170 mm (W × D × H) and a weight of 6.8 kg. It also seamlessly integrates thermal cycling, real-time scanning, and analysis software. The digiQuark was structured into three primary modules, the thermal cycling stage, optics, and PCB module. Additionally, it included a separate disk on which dPCR emulsion can be loaded for the PCR reaction to occur. This disk is a dedicated consumable containing 22,000 micro-wells and is referred to as the “22 k-single-disk”. The thermal cycling stage module was responsible for heating and cooling within the rotating disk during PCR reactions and includes components, such as the main heater, hot top heater (sub heater), motor, fan, and disk rotor (Supplementary Fig. 1). The optics module was designed to perform real-time high-speed fluorescent scanning during PCR reactions, allowing simultaneous scanning of up to five fluorophores (Supplementary Fig. 2). The PCB module served as the central control unit for the device (Supplementary Fig. 3). Sample partitioning, thermal cycling, and real-time scanning occurred in the 22 k-single-disk, which featured a robust sealing mechanism and efficient partitioning that minimized reagent loss (Supplementary Fig. 4). Notably, a crucial advantage of digiQuark is its simplicity in preparing dPCR emulsions, achieved through straightforward mixing of dPCR mixture and dedicated oil. This minimizes the potential for human error and contamination, as it does not require specialized techniques.

### Centrifugal force-based partitioning system

**Figure 2:**
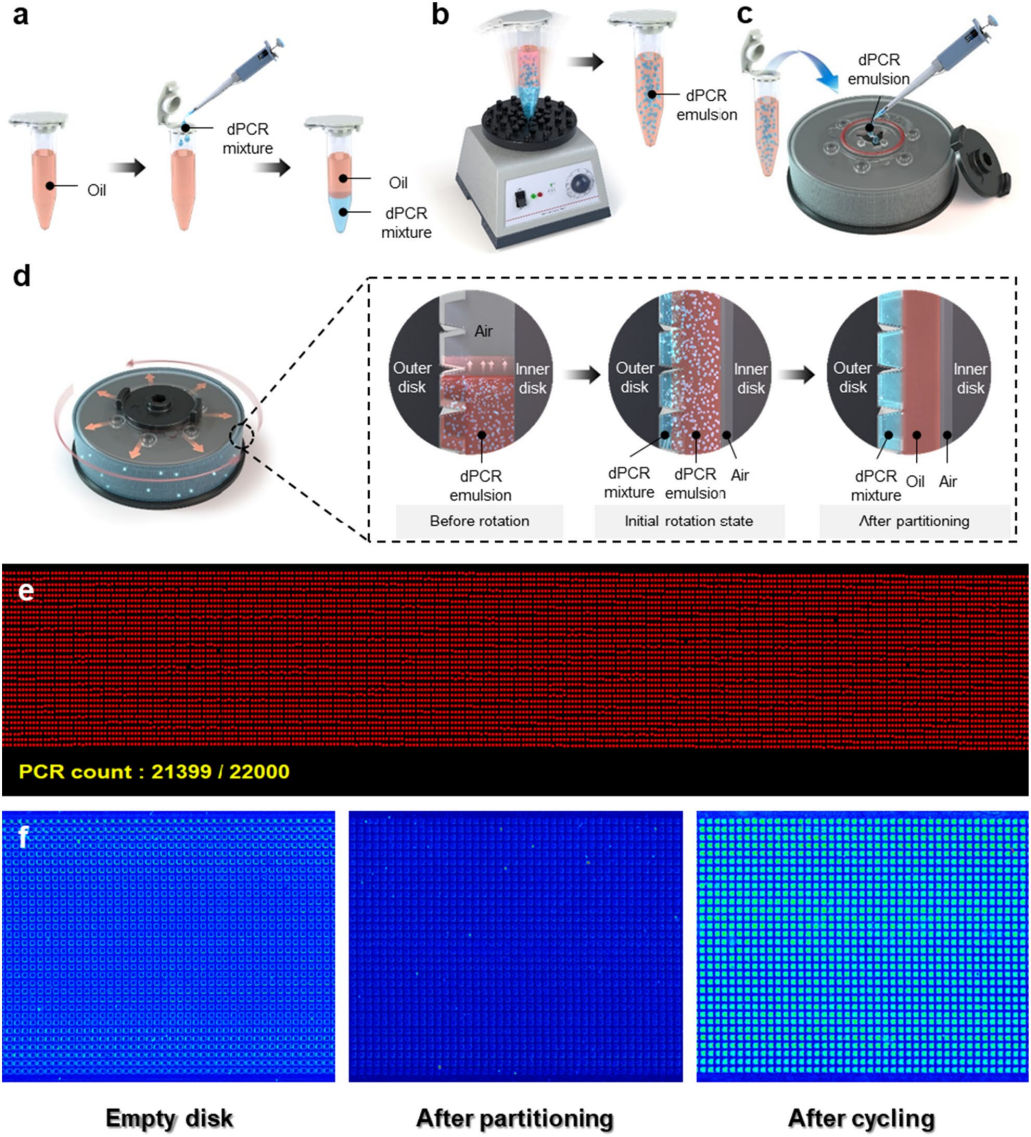
Schematic representation of the reagent partitioning process using centrifugal force. (**a**,**b**) Mixing of dPCR emulsion reagents. (**c**) Loading dPCR emulsion reagents into the 22 k-Single-Disk. (**d**) Schematic representation of partitioning the dPCR emulsion reagent as it is spun in the 22 k-Single-Disk. (**e**) Analysis of the partitioning ratio of all wells to total wells using metadata (21,399/22,000, 97.3%). (**f**) Scan images of the post-PCR cycle before reagent addition, after uniform partitioning, and after.

A cylindrical plastic injection disk was devised to partition the introduced samples using centrifugal forces. The disk comprised of (i) an inner disk that connects to the rotor, transmitting the rotational force of the motor to the reagent, (ii) an outer disk characterized by its intricate micro-patterns where centrifugally partitioned reagents are deposited, and (iii) a detachable disk cap designed for convenient reagent introduction and subsequent sealing (Supplementary Fig. 4). SEM analysis was performed to assess the volume uniformity of the micro-pattern applied to the external plate. The volume uniformity of the micro-pattern was determined to be 4.39% in 65 different patterns. The analysis results are detailed in Supplementary Fig. 5 and Supplementary Table 4. The mechanism by which the introduced reagents were uniformly distributed among the 22,000 micro-wells on the outer disk was driven by the dPCR emulsion and rotation. The dPCR emulsion is a reagent in which the dPCR mixture is evenly distributed within a specific oil (Fig. 2a and b). Furthermore, the oleaginous dedicated oil (3 mL) and the aqueous dPCR mixture (75 µL) were crucial in uniform partitioning. As the mixed dPCR emulsion rotates in the disk, stratification occurred due to the density difference between the dPCR mixture and dedicated oil. The denser aqueous dPCR mixture was distributed into the micro-wells, whereas the less dense oil performed an overlay function between the dPCR mixture and air layer; the overlay prevents cross-contamination between wells and evaporation of the dPCR mixture (Fig. 2c and d). In detail, the difference in density, which is a characteristic of the material, causes the dPCR emulsion to migrate outward from the rotation axis, as shown in the initial rotation state in Fig. 2d. After partitioning, these displayed emulsions can be allocated in each pattern as a dPCR mixture when the emulsion collapses owing to a thermal reaction, as shown after partitioning in Fig. 2d. The micro-wells could be scanned by the optics module, producing resulting images with a distribution rate of > 95% across all wells (Fig. 2e). The scans conducted pre-reagent introduction, post-partitioning, and post-PCR cycling revealed a consistent spread of the reagent, with amplification observed solely in the target-inclusive sections (Fig. 2f); this partitioning method minimized sample loss. Since each micro-well has a volume of 3.336 nL, the volume of all 22,000 wells is approximately 73.395 µL (Supplementary Fig. 5). Therefore, the reagent loss rate was only 2.14%, compared to the 75 µL volume of the utilized dPCR mixture.

### Comparison of end-point and true-positive select method results in digiQuark

**Figure 3:**
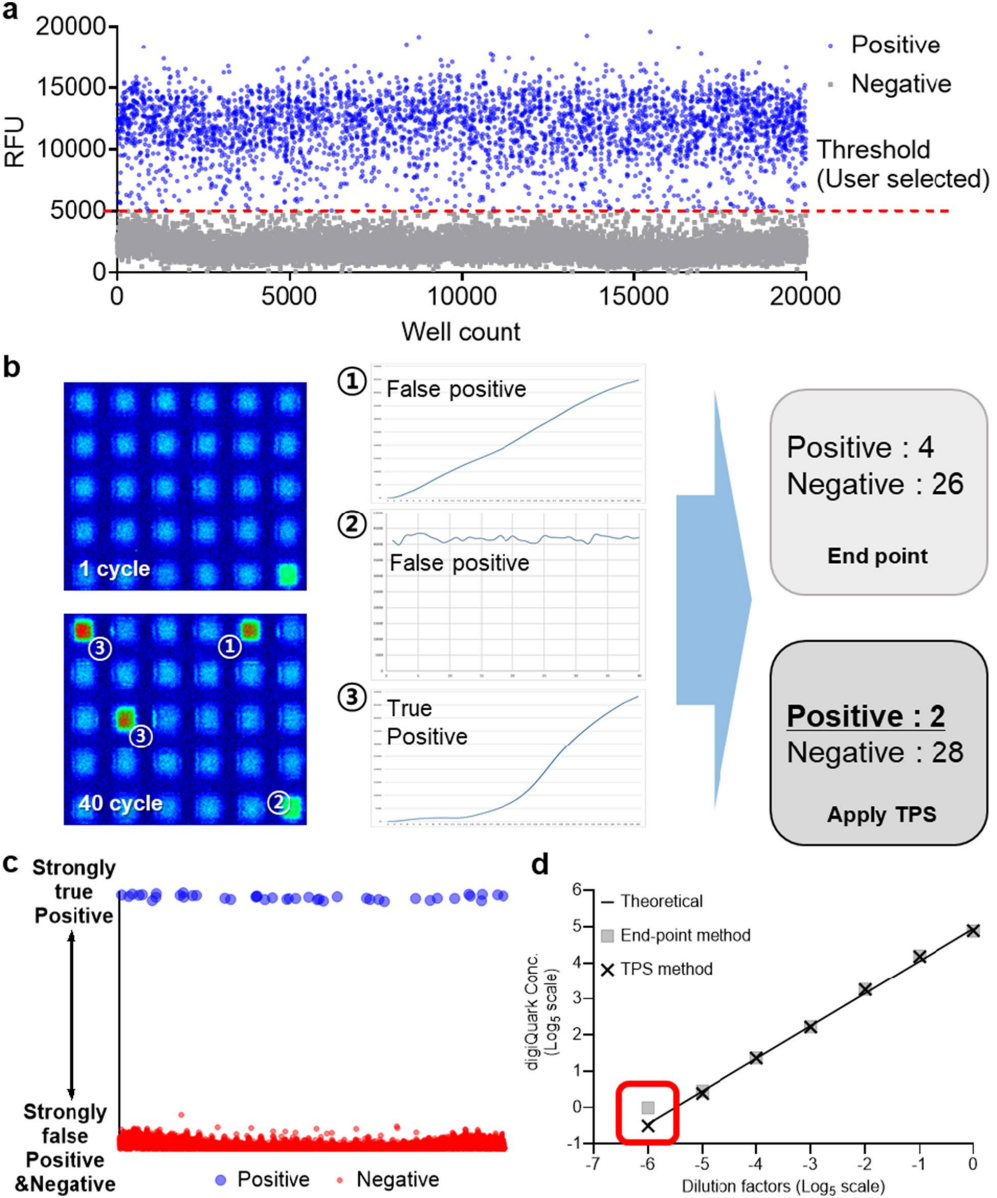
Comparison of results using end-point and proposed true-positive select(TPS) methods. (**a**) Threshold application graph of the conventional method. (**b**) Comparison of results using TPS and end point methods using digiQuark. (**c**) Rain problem improvement graph categorized by the TPS method. (**d**) Comparative analysis graph of the results of the two methods according to concentration.

A comparative analysis of digiQuark data was performed using end-point method, which is commonly used in conventional dPCR, along with a proposed new method called the true-positive select (TPS) method (Fig. 3). The conventional measurement method uses end-point detection and a user-definable threshold to distinguish between positive and negative wells^36^.When using this method, the value may vary depending on how the threshold is set owing to rain problem. Figure 3a shows threshold application to distinguish positive and negative wells. All wells with a high RFU based on the threshold were classified as positive. The TPS method selectively identifies only the true positives analyzed through ANN and applies them to Poisson’s distribution. This method can also select false positives that cannot be distinguished from the existing end-point method. Additionally, the TPS method addressed the rain problem using an approach where the positive/negative distinction becomes clearer (Fig. 3c) by the trained ANN. Further details regarding the ANN training are described in the method section. By applying the two methods to digiQuark, unlike end-point method, which tends to deviate from a linear graph as the concentration decreases on a log scale, the TPS method does not significantly deviate from a linear graph (Fig. 3d). While there was no significant difference between the end-point method and TPS methods in the intermediate concentration range (dilution factor 0 to − 4), the TPS method was closer to the theoretical data in distinguishing true positives in the low concentration range. This suggests that the TPS method improves the error of traditional analysis.

### Optimization for digiQuark

dPCR relies on the amplification of target DNA during thermal cycles; therefore, factors affecting amplification efficiency should be optimized. These factors include the cycling conditions for the given assay and the concentration and ratio of primers and hydrolysis probes^37,38^. Furthermore, to detect the specific optimal conditions for the digiQuark, the *EGFR* T790M mutation and wild-type copy number concentrations in 1 ng of genomic DNA from the NCI-H1975 cancer cell line were measured. This cell line carries the T790M (c.2369C > T) and L858R (c.2573 T > G) *EGFR* variants and the wild-type allele, which are amplified within the genome. The FAM probe targets the EGFR T790M mutant allele, and the Cy5 probe targets the wild-type counterpart.

**Figure 4:**
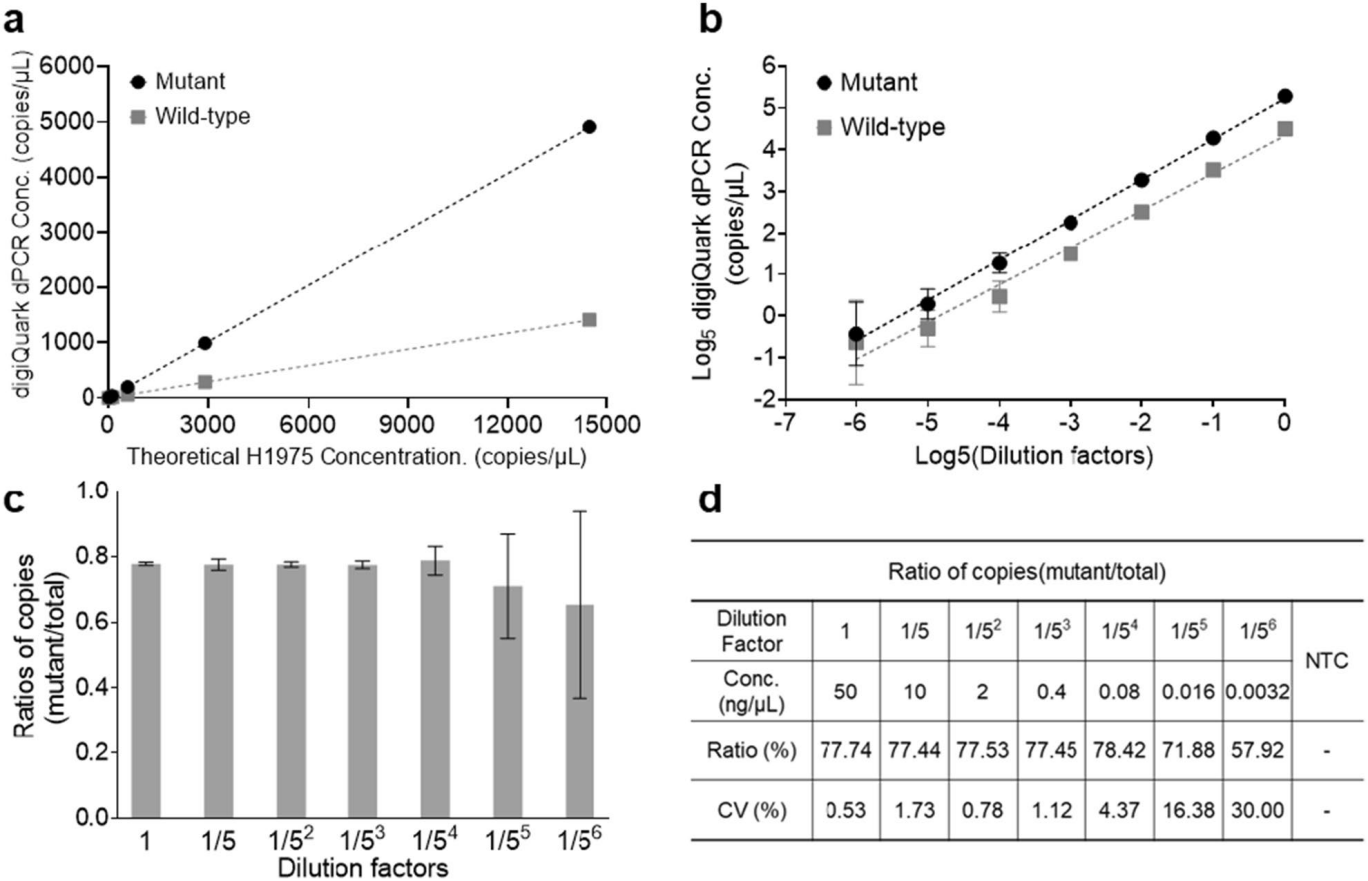
Optimization of digiQuark. (**a**) Linearity graph comparing calculated H1975 concentration (copies/µL) with the concentration values analyzed in digiQuark. (**b**) Linearity graph on a log scale based on concentration. (**c**,**d**) Ratio of copies and CV of mutant and wild-type for each concentration.

To optimize the cycling conditions for digiQuark, experiments were initially conducted using NCI-H1975 genomic DNA under conditions mimicking those of the QX200 system, which are as follows: initial condition at 93 ℃ for 600 s, denaturation at 94 ℃ for 30 s, and annealing at 58 ℃ for 30 s. However, these experiments revealed challenges, such as reagent evaporation and inconsistent partitioning (Supplementary Fig. 8). Iterative experiments involving adjustments of conditions showed the optimal conditions for the reactions to be the following: initial temperature range of 93–94 ℃ for 60 s, denaturation at 94–95 ℃ for 5 s, annealing at 52–55 ℃ for 5 s, and a scanning time of 35 s (Supplementary Fig. 8). The deviation from the existing T790M manual conditions can be attributed to the unique temperature measurement method and micro-well characteristics of digiQuark (Supplementary Fig. 6). Next, the performance of digiQuark was evaluated using dilutions of NCI-H1975 genomic DNA (50, 10, 2, 0.4, 0.08, 0.016, 0.0032 ng/µL) to test the accuracy based on the distribution and linearity of mutant (FAM fluorescence) and wild-type (Cy5 fluorescence). Experiments were conducted 30 times for each concentration and across different equipment setups (Fig. 4). TPS method was used to analyze copy numbers based on concentration; the linearity was 0.9999992 for the mutant type and 0.9999817 for the wild-type (Fig. 4a). Additionally, the linearity remained robust even on a log scale, with 0.99812 for the mutant type and 0.98517 for the wild-type at a concentration of up to 0.0032 ng/µL, confirming the optimization of the equipment (Fig. 4b). The ratio of copy numbers for the mutant and wild types based on concentration using the TPS method is presented in Fig. 4d and e. As the concentration decreased, the variation increased. Notably, starting from 5^–5^ (0.016 ng/µL), a significant surge was observed in the ratio variation. However, from 1 (50 ng/µL) to 5^–4^ (0.08 ng/µL), the ratios were consistently maintained at 77–78%. Moreover, evaluation was conducted under conditions of mutant ratios of 2, 1, and 0.2%, and the linearity according to the ratio was confirmed to be at the level of 0.9994 (Supplementary Table 5).

### Comparison of three dPCR methods using copy number values, linearity, and sensitivity of EGFR variants quantification

**Figure 5:**
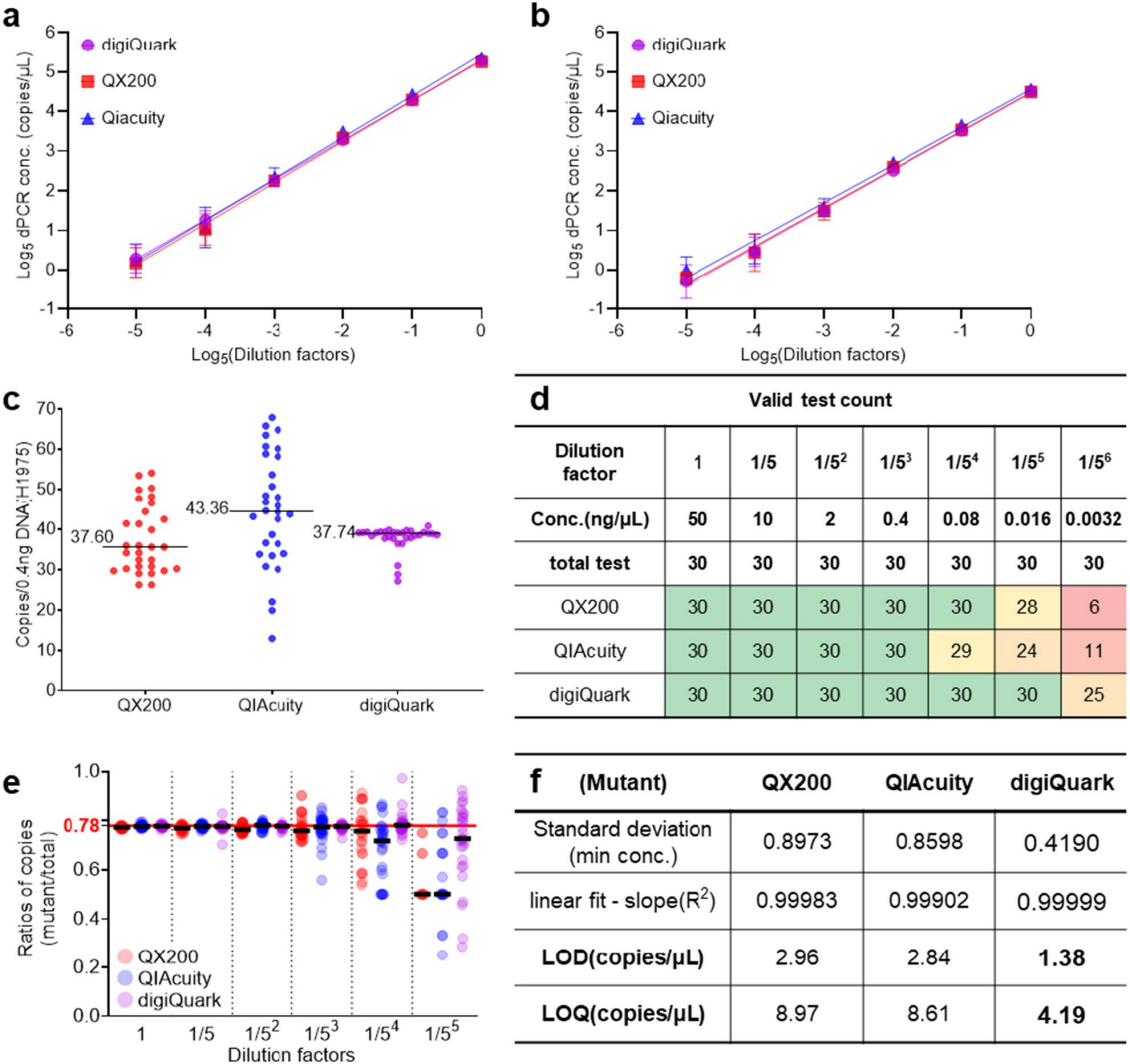
Comparative analysis of DNA (H1975) concentration across platforms. (**a**,**b**) Platform-specific log-scale graphs depicting mutant/wild-type linearity based on concentration. (**c**) Copy number analysis of the mutant at 0.4 ng/µL across platforms. (**d**) Effective test counts relative to the total tests based on concentration. (**e**) Mutant/total copy ratio across platforms based on concentration. (**f**) Platform-specific LOD and LOQ analysis for the mutant.

In order to compare the three dPCR platforms, the copy number concentrations of the EGFR variants were analyzed. The templates used were cancer cell lines NCI-H1975. When genomic DNA from NCI-H1975 was analyzed using the T790M, the linearity of the measured copy numbers based on dilution for the QX200, QIAcuity, and digiQuark platforms were 0.99798, 0.99650, and 0.99996 for the mutant (Fig. 5a), and 0.99640, 0.99300, and 0.99834 for the wild-type (Fig. 5b), respectively. This underscores that all three platforms exhibited similar performance levels. When evaluating the copy numbers across platforms at the same concentration, a concentration of 0.4 ng/µL showed readings of 37.6, 43.36, and 37.74 copies/µL for QX200, QIAcuity, and digiQuark, respectively (Fig. 5c). Conversely, QIAcuity slightly surpassed the others in copy numbers, these figures are within the data variance, indicating no significant deviation among the instruments. However, performing 30 repeated tests for each concentration across the platforms revealed instances where no readings were detected at low concentrations. Taking the 5^–6^ concentration as a benchmark, the QX200, QIAcuity, and digiQuark registered measurements 6, 11, and 25 times respectively. Comparatively, digiQuark exhibited a higher efficacy rate in these low-concentration experiments (Fig. 5d). Further analysis of the mutant/wild-type ratio across platforms revealed that QX200 and QIAcuity deviated significantly from the average ratio with increasing variations starting from concentration 5^–4^. Conversely, digiQuark maintained a stable variation and average ratio up to concentration 5^–4^; however, it exhibited a significant deviation from concentration 5^–5^ onward. (Fig. 5e). To compare the analytical sensitivity of QX200, QIAcuity, and digiQuark, the limit of detection (LOD) and limit of quantitation (LOQ) for each platform were further evaluated (Fig. 5f). The data pertaining to each dataset and standard deviation (SD), coefficient of variation (CV), and mean are documented in Supplementary Table 3. The LOD and LOQ values for QX200 were established at 2.96 copies/µL and 8.97 copies/µL, respectively, correlating with concentrations of 0.01 ng and 0.03 ng of NCI-H1975 genomic DNA. Regarding QIAcuity, the LOD and LOQ were 2.84 and 8.61 copies/µL, equivalent to the concentrations of 0.01 ng and 0.03 ng. digiQuark showed a LOD and LOQ at 1.38 and 4.19 copies/µL, correlating with the concentrations of 0.005 ng and 0.015 ng, respectively. The LOD and LOQ analysis revealed that the values of digiQuark were approximately half of the QX200 and QIAcuity platforms. Notably, similar precision levels were observed across the platforms at higher concentrations; the digiQuark exhibited superior accuracy when utilizing minimal template quantities. In conclusion, for the analysis of the EGFR T790M mutant, the digiQuark platform outperformed the other platforms; it showcased enhanced sensitivity and accuracy at lower concentrations.

## Discussion

Accurate nucleic acid measurement is essential for the diagnosis of trace amounts of cancer genes and infectious diseases, such as COVID-19. As the most advanced technology available for nucleic acid detection and quantification, dPCR is being used in the development of sever techniques applicable in a wide range of fields. However, challenges, including the rain problem, liquid loss, false positives, and hard handling of the equipment, limit its application. To mitigate these challenges, a new dPCR device incorporating novel technologies and boasting superior sensitivity, was developed, and its performance was compared with the liquid-based QX200 and nanoplate-based QIAcuity. The characteristics of each equipment compared are summarized in Table 1.

**Table 1:** Comparison of characteristics of each dPCR equipment.

	QX200 system	QIAcuity	digiQuark
System description			
Number of modules	3*	1	1
Instrument dimension (mm)	Generator: 280 × 360 × 130	380 × 450 × 650	185 × 395 × 170
	Reader: 660 × 520 × 290		
On-board computer	No	No	No**
Partitioning type	ddPCR (droplet digital PCR)	nanoplate dPCR	crdPCR (centrifugal force real-time digital PCR)
	Droplet (oil emulsions)	Solid (nanoplate)	Physical (centrifugal force)
Data type	end-point	end-point	Real-time, end-point
Number of partitions	20,000	8500 or 26,000	22,000
Fluorescent channels	1–2	1–5	1–5
Adapted to single or series	1–96 samples/run	8–96 samples/run	1 sample/run***
Workflow			
Total time (min)	180	150	120
Hans on time (min)	30	< 15	< 5
Technical steps	6	3	2
Ease of use	+	+ +	+ + +

*Including PCR thermo-cycler.

**The next version has an on-board computer.

***The next version has 8 samples/run.

The primary features of the device used were as follows: (i) centrifugal distribution of the sample in a 22 k-Single-Disk, (ii) a unique dPCR emulsion composition to prevent evaporation during amplification and a distinct heating method, and (iii) a novel analysis method (TPS) applied for real-time scanning and AI-driven false positive screening, enhancing sensitivity. The 22 k-Single-Disk, manufactured through injection molding of cylindrical plastic, employed centrifugal force to dispense the sample. Using the hot stamping technique, consistent micro-wells (22,000 wells) were created inside the outer 22 k-Single-Disk; these wells, similar in number to other devices, can accommodate a larger volume (up to 3.336 nL) than other machines. Furthermore, two fluorophores, FAM and Cy5, were used in the experiments; however, the optic module of the device used could scan up to five fluorophores, allowing diverse sample analysis using matching probes^39,40^. Additionally, adjusting the Single-Disk size could yield over 100,000 micro-wells per Single-Disk. A notable feature when using this 22 k-Single-Disk was the sample preparation could be completed via simple vortexing. After generating the dPCR emulsion, it has to be inserted into the inlet of the 22 k-Single-Disk, sealed with a custom cap, and mounted onto a rotor; this process requires no special manipulation, making it easy to operate. This simplified method minimizes the chances of human error and prevents potential contamination.

The dPCR emulsion introduced into the 22 k-Single-Disk was dispensed into individual micro-wells by centrifugal force. The oil separated from the emulsion moved to the upper layer of the dPCR mixture due to the difference in density, preventing evaporation. Additionally, the oil layer prevented potential cross-contamination among the wells during amplification. Temperature control for the amplification of the dPCR mixture was performed in the heater module (Supplementary Fig. 1). The 22 k-Single-Disk was heated through the main heater located inside the rotor that secures the 22 k-Single-Disk, and the hot top situated above the 22 k-Single-Disk prevented evaporation caused by temperature variations inside and outside the 22 k-Single-Disk. Furthermore, an indirect heating and temperature measurement method was applied to regulate the temperature of the rotating 22 k-Single-Disk without directly heating the dPCR mixture. To enhance the accuracy of temperature measurements for the dPCR mixture, thermal structural analysis simulations were performed. In addition to calibration through thermal analysis, the precision of temperature calibration for the internal reagents was significantly improved by cross-referencing with a specialized temperature probe that emits a specific intensity of luminescence at specific temperatures (Supplementary Fig. 6). The cooling mechanism involved drawing external air from the top of the 22 k-Single-Disk via a fan located below the rotating section; the inhaled external air circulated from the 22 k-Single-Disk to the fan, cooling the 22 k-Single-Disk in the process. The overall amplification took approximately 2 h for a 40-cycle reaction, with a ramping cycle time of 3 min/cycle. This is faster than the 2.5 h required by QIAcuity and significantly quicker than QX200's thermal cycling and reading times, thereby highlighting the efficiency of digiQuark. Furthermore, the cooling mechanism of digiQuark requires no additional system, offering compact advantage.

Conventional dPCR devices rely on end-point scans and Poisson's distribution, which have limitations, including compromised sensitivity at low concentrations, which can generate false positives and variations in end-point relative fluorescence unit (RFU), ultimately resulting in the rain problem. However, the novel approach implemented in the present study obtained individual well real-time PCR graphs based on real-time scanning during the amplification process. By selecting results based on the real-time graph and leveraging AI algorithms, false positives were eliminated, and only true positives were retained. This enhanced sensitivity and enabled the detection of lower concentrations, proving twice as sensitive as other devices (Fig. 5f). Furthermore, digiQuark consistently measured with an 83% success rate (25/30 attempts) even at a 1/5^6^ dilution (Fig. 5d).

The recent global pandemic caused by the SARS-CoV-2 virus underscores the increasing importance of detecting low concentrations of DNA or RNA. Current dPCR devices, despite their potential, grapple with challenges, such as intricate experimental procedures, and variable outcomes based on operator skill. However, the innovative dPCR device, namely digiQuark, simplifies the process in a compact unit. Its ability to detect nucleic acids at low concentrations renders it a vital tool for early cancer detection. Furthermore, these technological advancements will expedite the development of next-generation PCR devices focused on absolute quantitative analysis.

## Method

### Thermal cycling system module

The heating system of the thermal cycling system module comprises an inner main heater located within the disk rotor and a hot-top heater positioned above the 22 k-Single-Disk (Supplementary Fig. 1a). This system controls heating within an accuracy range of ± 0.5 ℃, and the heating ramping rate was set at 0.58 ℃/s. The main heater, structured in a ring formation inside the disk rotor, elevates the temperature of the rotating 22 k-Single-Disk. The hot-top heater, shaped in a circular design at the top of the 22 k-Single-Disk, prevents the evaporation of reagents within the 22 k-Single-Disk.

The air cooling system comprises a main fan located at the bottom, which induces the intake of air from the exterior through upper vents. As the introduced air passes over the surface of the disk sub-unit, heat exchange occurs, and the air is eventually expelled through the main fan at the base (Supplementary Fig. 1b). Through this process, ambient temperature circulation facilitated a decrease in temperature, and the cooling ramping rate was maintained at 0.50 ℃/s.

### Optics module

The optics module comprises an emitter, detector, and optical component, and it reads the fluorescence signals by moving vertically at a slow pace over the rotating micro-wells during the amplification of the samples. The optics module consists of 1–5 independent measurement modules (Supplementary Fig. 2, Supplementary Table 1), capturing fluorescence signals after every annealing phase in each cycle and generating two-dimensional images and data maps. Within the sample 22 k-Single-Disk, the inner walls of the micro-wells each captured signals with a minimum resolution of 5 µm; the signals were mapped based on their respective positions, culminating in a two-dimensional image.

### PCB module system

The PCB module system is designed to be centered around FPGA, enabling synchronized control of various functions in real-time (Supplementary Fig. 3); it regulates heating and cooling through the operation of the heating system module and fans. Simultaneously, it employs an IR temperature sensor to monitor the temperature of the 22 k-Single-Disk, ensuring it is maintained at the appropriate temperature. During light measurements, fluorescence signals applied to the photodiode are converted into digital signals, which are transmitted to a PC using a USB protocol. For precise fluorescence measurements, the module controls signal from the light measurement section, which consists of a laser diode (LD), multi-pixel photon counter (MPPC), and an amplifier.

### Development of micro-well 22 k-single-disk

The 22 k-Single-Disk containing micro-wells was fabricated using plastic injection molding in a cylindrical shape to distribute samples using centrifugal force (Supplementary Fig. 4). The inner 22 k-Single-Disk incorporated a ribbed shape to prevent deformation and evaporation within the 22 k-Single-Disk due to pressure variations during PCR. Furthermore, it was made with a matte black finish to avoid generating noise data during optical module readings. The outer 22 k-Single-Disk was crafted from highly transparent polycarbonate material, which is vital for measuring emitted fluorescence from the reagents. The inside of the outer 22 k-Single-Disk included a micro-well pattern produced via the hot stamping method. The employed micro-well pattern for the experiments measured 200 × 200 µm with a depth of 100 µm, totaling 22,000 wells (Supplementary Fig. 5). To ensure that the assembly of the inner and outer 22 k-Single-Disks is leak-proof during tests, the ultrasonic welding technique was used to assemble them. The 22 k-Single-Disk cap featured a silicone O-ring to prevent pressure loss from internal heat during PCR and was designed for easy sealing after reagent introduction.

### Temperature control

The digiQuark employs an indirect heating approach instead of directly heating the loaded reagent in the 22 k-Single-Disk. Additionally, temperature measurements of the reagent were indirectly conducted using an IR sensor. Consequently, there was an inherent discrepancy between the actual reagent temperature and the temperature recorded by the IR sensor; to compensate for this discrepancy, a thermal analysis simulation was conducted for the entire structure. Based on the design specifications, a thermal analysis spanning from the internal heater ring, inner air layer, rotor layer, inner 22 k-Single-Disk layer, outer air layer, oil layer, and reagent layer to the outermost layer was performed. The temperature of the outer layer was directly measured using an IR sensor (Supplementary Fig. 6a and b). For the thermal analysis, finite element method (FEM) was employed using COMSOL Multiphysics 6.0. For precise calibration, a specialized temperature probe that emits specific luminescence at specific temperatures was employed (Supplementary Fig. 6c). The temperature of the internal reagent was inferred based on the emissivity of the 22 k-Single-Disk material and the thermal conductivity from the internal heater to the outer surface of the 22 k-Single-Disk. This was calibrated to allow temperature-dependent analysis consistent with the results verified using a temperature probe reagent.

### Optimization and performance evaluation of the developed equipment

#### Preparation of genomic DNA

NCI-H1975 (ATCC) cells were grown in RPMI 1640 media (Cytiva, Uppsala, Sweden) with 25 mM l-Glutamin (Cytiva, Uppsala, Sweden) supplemented with 10% FBS (Cytiva, Uppsala, Sweden) and 1% Penicillin–Streptomycin (Cytiva, Uppsala, Sweden) at 37 ℃, 5% CO_2_. Next, 1 × 10^6^ cells were prepared as pellets to extract genomic DNA using a Maxwell^®^ RSC Genomic DNA Kit (Promega, USA) for the Maxwell^®^ RSC 48 (Promega, USA). The extracted genomic DNA was eluted using the Elution Buffer in the Maxwell® RSC Genomic DNA Kit, and the concentration was measured using a Quantus™ Fluorometer (Promega, Madison, WI, USA).

#### Primer and probe design

Hydrolysis probe-based assays were designed for the target, namely EGFR (Supplementary Table 2). The final designed primer and probe were forward primer, reverse primer, mutant probe (FAM), and two wild-type probes (HEX, Cy5).

#### Analysis using the QX200 (Droplet dPCR, ddPCR)

Experiments were conducted with a QX200 Droplet Digital PCR system (Bio-Rad, Hercules, CA). PCR reactions were prepared in a final volume of 20 µL that contained 10 µL of Supermix for Probes (no dUTP; Bio-Rad) and 3 µL of the assay for *EGFR* (10 pmol forward primer, 10 pmol reverse primer, 5 pmol FAM probe, and 5 pmol HEX probe per reaction). For each reaction, a non-template control (NTC) was included. A QX200 Droplet Generator (Manual DG, Bio-Rad) or Automated Droplet Generator (Auto DG, Bio-Rad) was used to generate the droplets. PCR was performed in a T100 Thermal Cycler (Bio-Rad). The reaction cycle was as follows: 10 min at 95 ℃, followed by 45 cycles of 30 s at 95 ℃ and 1 min at 58 ℃. After amplification, the plate was loaded onto the QX200 Droplet Reader (Bio-Rad) and analyzed using QuantaSoft Software version 1.7.4. (Bio-Rad). All of the thresholds were set up manually to allow the distinction between positive and negative droplets. Only the reactions with more than 15,000 valid droplets were used for analysis.

#### Analysis using the QIAcuity (nanoplate-based dPCR, ndPCR)

Experiments were conducted with a QIAcuity Digital PCR system (Qiagen, Hilden, Germany). PCR reactions were prepared in a final volume of 40 µL that contained 10 µL of 4 × probe PCR Master Mix (Qiagen) and 9.6 µL of the assay for *EGFR* (10 pmol forward primer, 10 pmol reverse primer, 10 pmol FAM probe, and 10 pmol Cy5 probe per reaction). The mixture was prepared in a pre-plate and then transferred into the 24-well 26 K Nanoplate and sealed with the Nanoplate seal. For each reaction, an NTC was included. PCR was performed using QIAcuity One, 5plex System in QIAcuity Nanoplate 26 K, 24-well (Qiagen); the reactions conditions were as follows: 10 min at 95 ℃, followed by 45 cycles of 30 s at 95 ℃ and 1 min at 58 ℃, and a final imaging step by reading in the FAM, Cy5 channel. The analysis was performed using QIAcuity Software Suite version 2.2.0.26 (Qiagen). All of the thresholds were set up manually to allow the distinction between positive and negative partitions. Only the reactions with more than 25,000 valid partitions were used for analysis.

#### Analysis using the digiQuark (spinning dPCR, sdPCR)

The digiQuark is an instrument that allows partitioning, thermal cycling, and detection on a single instrument. digiQuark uses a two-step process to prepare the dPCR emulsion. First, PEG-10 Dimethicone (Dow Chemical, USA; 2 ~ 4 wt%) was added to dimethyl silicone oil (ShinEtsu chemical, Japan) and mixed for 5 min at 3000 rpm using a vortex. After the initial mixing, 75 µL of PCR mixture was added to 3 mL of silicon oil and mixed for 1 min at 3000 rpm using a vortex to prepare dPCR emulsion. The 75 µL of PCR mix included 18.75 µL of 4 × qPCR master mix (Elpisbiotech, Daejeon, Republic of Korea) and 18 µL of the assay for *EGFR* (10 pmol forward primer, 10 pmol reverse primer, 10 pmol FAM probe and 10 pmol Cy5 probe per reaction).

The reaction mixture was loaded into the 22 k-Single-Disk and spread evenly within the 22 k-Single-Disk via centrifugal force (3600 rpm). The PCR reactions for *EGFR* were conducted under the conditions of 1 min at 95 ℃ and 45 cycles of 5 s at 95 ℃ and 5 s at 58 ℃. Only the reactions with more than 21,600 valid micro-wells were used for analysis.

### Data analysis for digiQuark

#### Real-time scanning

The sample-loaded 22 k-Single-Disk rotated counterclockwise, and the optical system moved from the bottom to the top of the 22 k-Single-Disk via linear stage motion, acquiring light signals. The point marked inside the rotating rotor, detected by a photo sensor, was designated as the origin at which data is captured. Scanning commenced from the designated origin in the direction of rotation, and two scanning resolutions were selected. The minimum resolution was 5 µm per pixel at 3600 rpm, with a data capture rate of approximately 2.3 million samples/s. In this experiment, the condition of 10 µm per pixel at 3600 rpm was used, with a sampling speed of about 1.2 million samples/s. The scanning data of the cylindrical rotating 22 k-Single-Disk was acquired in a helical direction. As the images acquired in this helical manner fundamentally followed the shape of the cylindrical 22 k-Single-Disk, the data was stored based on cylindrical coordinates when cropped using the photo sensor's origin. An algorithm that converts the cylindrical coordinate data into Cartesian coordinates was applied to obtain the 2D image raw data. Each sample was saved in 16 bits, represented as an unsigned integer; the RFU had a value range of 0–65,535. Additionally, to enhance readability, the grayscale RFU was transformed to an RGB scale through pseudo-color mapping for display^41^.

#### Data analysis

Using the scanned data, the 2D image raw data was referenced to track the positions of the micro-wells, securing meta-data by averaging the data from the center 5 pixels of each well. An experiment is deemed valid only if the secured meta-data has over 21,000 valid wells. Through the meta-data, a real-time graph was derived for each micro-well, acquiring the relative fluorescence unit (RFU) for each cycle. To distinguish between true/false positives from the real-time PCR data for each well was obtained as a benchmark for each well; this standard was employed using a single-layered Perceptron ANN (Artificial Neural Network) model^42^ (Supplementary Fig. 7).

1 $${y}_{\_in}=bias+\sum_{i=1}^{40}{x}^{i}{w}^{i}.$$

The activation function for the Perceptron is given in the equation below.

2 $$f\left({y}_{{}_{in}}\right)=\left\{\begin{array}{c}1 if\,\, {y}_{{}_{in}}>0\\ -1 { if\,\,R y}_{{}_{in}}\le 0\end{array}\right..$$

The RFU values of 40 cycles were used as training set. A true positive response was set to target (t) 1, and a false positive or false negative response was set to target (t) –1. Each set of 100 was trained to generate weights (w) with a learning rate (α) of 0.5, and the training method used is as shown in Eqs. (3)–(7).

3 $$if \,\,f\left({y}_{\_in}\right)\ne t,$$

4 $${w}_{i}\left(new\right)={w}_{i}\left(old\right)+\alpha t{x}^{i}, i=1\sim 40,$$

5 $$else bias\left(new\right)=bias\left(old\right)+\alpha t,i=1\sim 40,$$

6 $${w}_{i}\left(new\right)={w}_{i}\left(old\right), i=1\sim 40,$$

7 $$bias\left(new\right)=bias\left(old\right),i=1\sim 40.$$

True positive well data were selected using the real-time graph-based Perceptron ANN above. Subsequently, Poisson's distribution was applied based on the selected true positive well data, and this method was defined as the TPS method. The Poisson distribution follows the equations given in reference^43^.

8 $${C}_{dPCRmixture}=-ln\left(1-\frac{{N}_{P}}{{N}_{T}}\right)\cdot \frac{{10}^{3}}{{V}_{P}},$$

where N_P_ represents the number of positive wells, N_T_ represents the total number of wells, and V_p_ is the volume of a single well, presented in nanoliters (nL).

9 $$LOD=3.3*SD/Slope,$$

10 $$LOQ=10.0 \times SD/Slope,$$

The measurement limitations and performance analysis of the equipment employed the LOD and LOQ methods^44–49^. LOD and LOQ are determined using the formulas above, where 22 k-Single-Disk represents the standard deviation at the lowest measurable concentration for each device, and slope utilizes linearity up to the lowest measurable concentration. The data used to derive each value was in units of copies/µL.

## Supplementary Information

Supplementary Information.

## Data Availability

The sequence data generated and analyzed during the current study can be accessed online at https://docs.google.com/spreadsheets/d/1n9T4-5ECPY5SCCENvi6MA9SjR7yXa6EW/edit?usp=sharing&ouid=107752273101422534587&rtpof=true&sd=true also the another datasets used and analyzed during the current study available from the corresponding author on reasonable request.
