# Supplementary material for: Developing centrifugal force real-time digital PCR for detecting extremely low DNA concentration
Source: Sci Rep. 2024 May 21;14:11522. doi: 10.1038/s41598-024-62199-5 (PMC11636855; doi:10.1038/s41598-024-62199-5)
Supplement: Supplementary file 1 — Supplementary Information. [file 41598_2024_62199_MOESM1_ESM.docx]

**[Supplementary Information]**


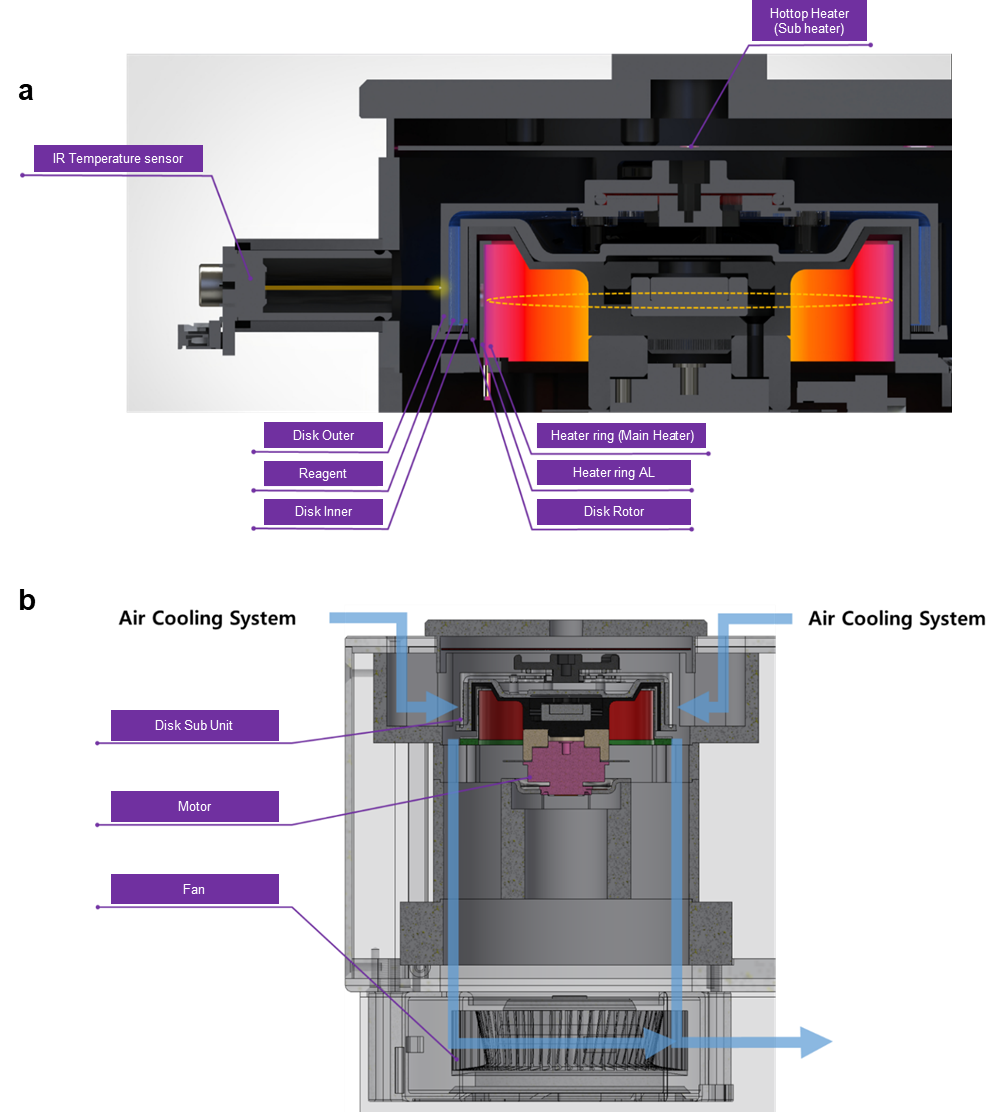


**Supplementary Fig. 1**: **Thermal cycling stage module**. Schematic diagram of **a,** heating system and **b,** air cooling system.


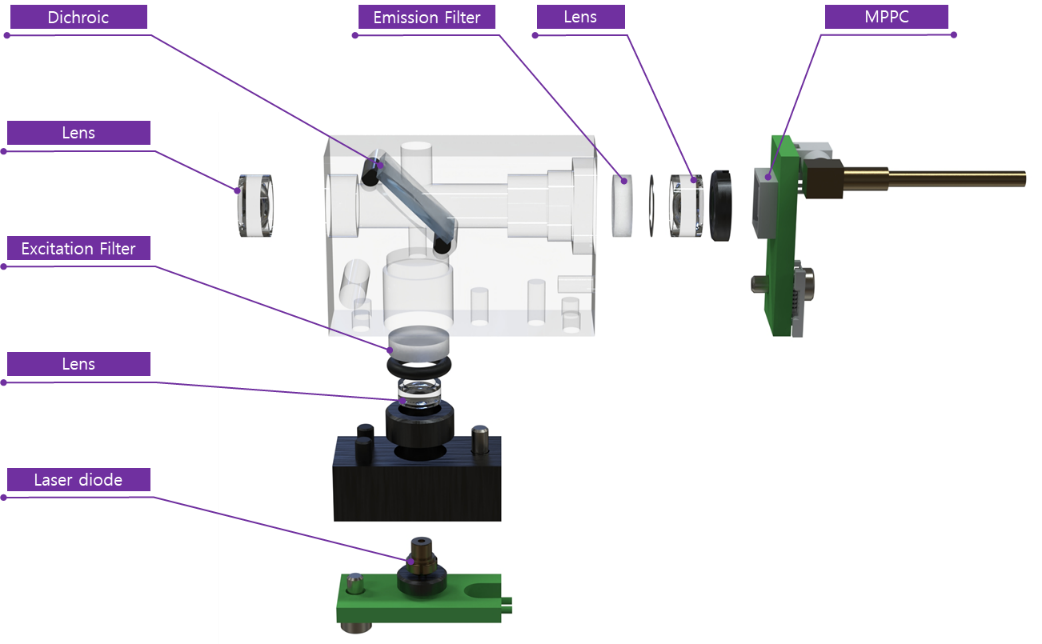


**Supplementary Fig. 2**: **Schematic diagram of optics system module**. The optics system module consists of a LASER diode, lens, excitation/emission filter, dichroic mirror, and MPPC.


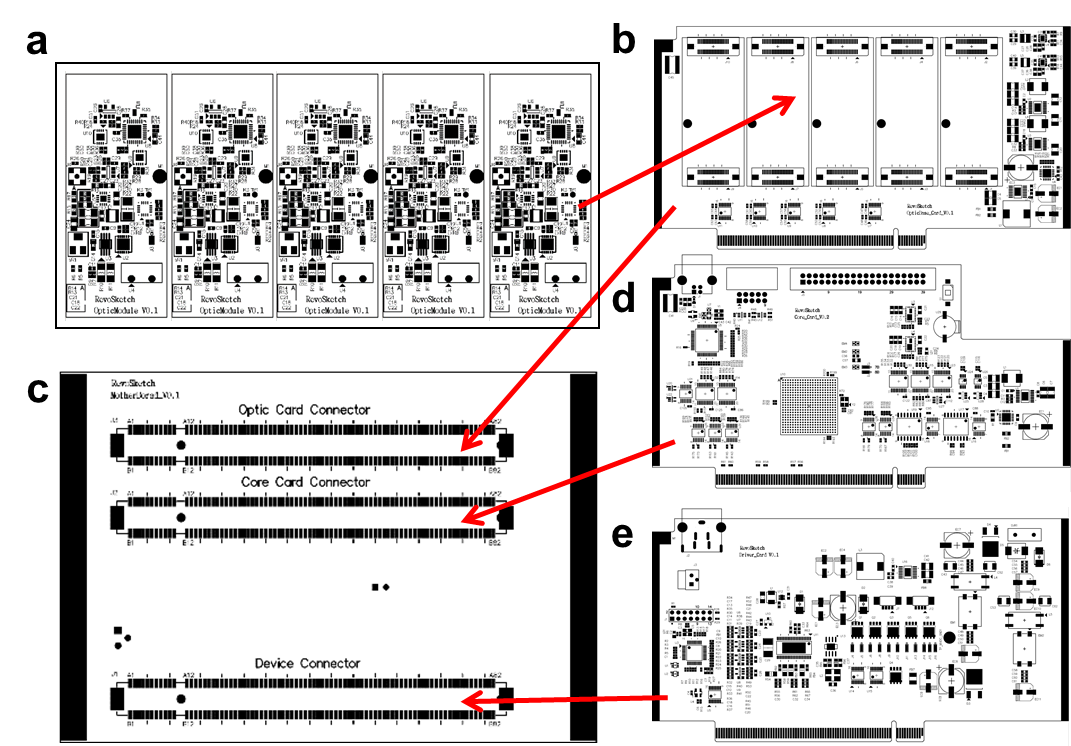


**Supplementary Fig. 3**: **PCB module**. **a,** Optic module card. **b,** Optic base card. **c,** Motherboard. **d,** Core card. **e,** Device card.


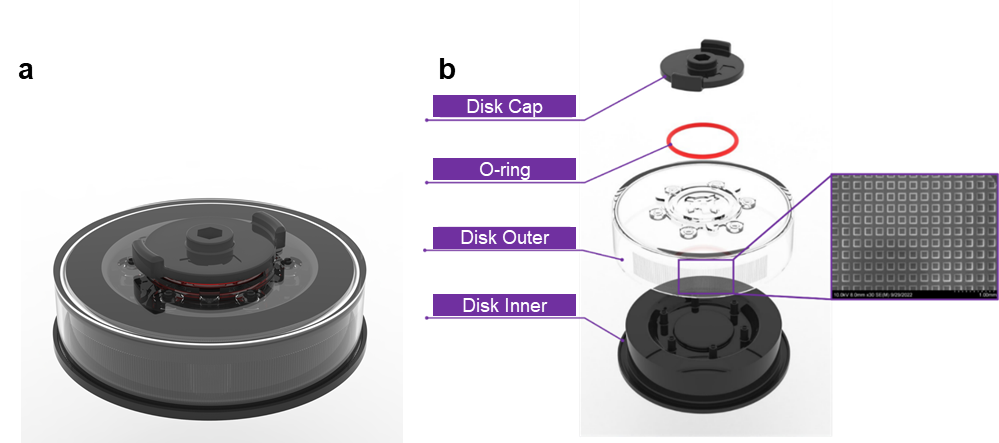


**Supplementary Fig. 4**: **Micro-well dish(22k-Single-Disk, SD) consists of a disk cap, an O-ring, the inner disk, the outer disk, and a micro-well pattern**.

**Supplementary Fig. 5**: **Micro-well Volume Measurement**. **a**, SEM image of the micro-well in the outer disk produced by the hot stamping method. **b**, Schematic of the micro-well pattern of the hot stamping mold and its volume measurement results.

**Supplementary Fig. 6**: **Temperature calibration of digiQuark**. **a**, Thermal-structure analysis conditions through FEM simulation. **b**, Results of the temperature gradient graph obtained from the thermal conduction simulation. **c**, Melting curve analysis using a 57 °C temperature probe.

**Supplementary Fig. 7**: **Schematic diagram of the True/False positive distinguished method using ANN**. True/false positive distinguished are according to the decision conditions in the hidden layer.


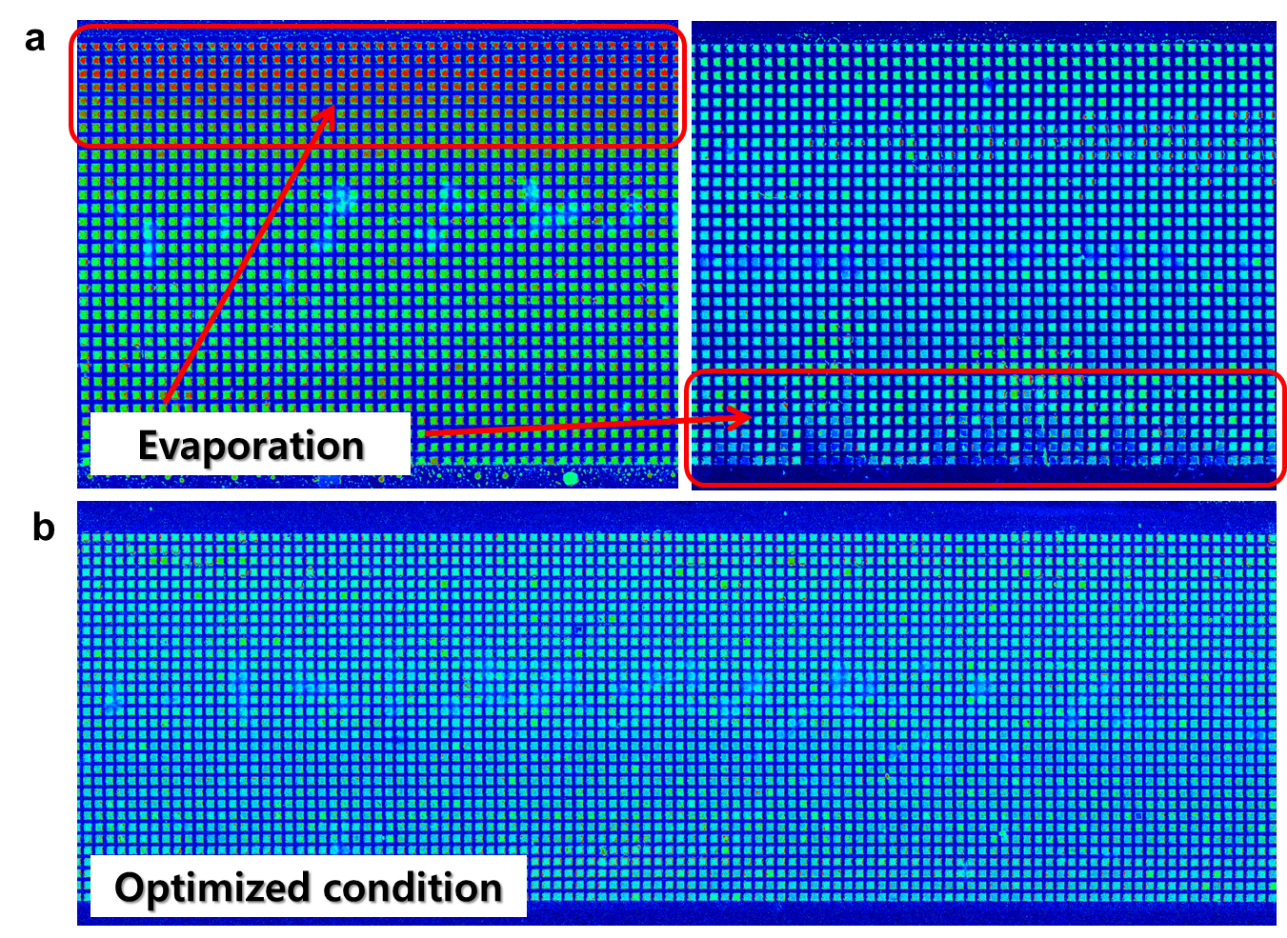


**Supplementary Fig. 8**: **Evaporation behavior based on thermal cycle temperature conditions**. **a**, Evaporation due to temperature condition mismatch. **b**, PCR image under digiQuark's optimized temperature conditions.

**Supplementary Table 1. Information on each optics module**

|  | **Fluorescent target** | **LASER Diode** | **Excitation wavelength** | **Emission wavelength** |
| --- | --- | --- | --- | --- |
| **F1** | FAM | Blue LASER Diode | 493 nm | 517 nm |
| **F2** | TET | Green LASER diode | 521 nm | 542 nm |
| **F3** | ROX | Green LASER diode | 555 nm | 569 nm |
| **F4** | Texas Red | Green LASER diode | 586 nm | 603 nm |
| **F5** | Cy5 | Red LASER diode | 651 nm | 670 nm |

**Supplementary Table 2. Sequences of primers and probes**

|  | **Name** | **Sequences** |
| --- | --- | --- |
| **Primer** | Forward | 5’-CATCTGCCTCACCTCCAC-3’ |
|  | Reverse | 5’-ACCAGTTGAGCAGGTACTGG-3’ |
| **Probe** | Mutant-FAM | 5’-[FAM]-CATCATGCAGCTCATGCCC-[BHQ1]-3’ |
|  | Wild-type-HEX | 5’-[HEX]-CATCACGCAGCTCATGCC-[SFCQ]-3’ |
|  | Wild-type-Cy5 | 5’-[Cy5]-CATCACGCAGCTCATGCC-[SFCQ]-3’ |

**Supplementary Table 3. 3-platform dPCR result**

| **QX200 (mutant)** | | | | | | | |
| --- | --- | --- | --- | --- | --- | --- | --- |
| **[copies/μL]** | 5^0^ | 5^-1^ | 5^-2^ | 5^-3^ | 5^-4^ | 5^-5^ | 5^-6^ |
| **stdev** | **168.833** | **57.0717** | **28.6174** | **8.42536** | **2.84771** | **0.89731** | **0.80051** |
| **avg** | **4669.21** | **992.09** | **214.106** | **37.6047** | **5.19458** | **1.3244** | **0.0994** |
| **1** | 4547.457 | 912.109 | 187.3869 | 30.81055 | 4.715448 | 1.988233 | ND |
| **2** | 4561.296 | 848.8992 | 180.7649 | 26.23887 | 3.595204 | ND | ND |
| **3** | 4555.362 | 952.2133 | 201.5665 | 29.71696 | 1.358433 | 0.650937 | ND |
| **4** | 4556.818 | 1013.764 | 184.8285 | 34.17445 | 1.998817 | 0.598423 | ND |
| **5** | 4534.804 | 972.4793 | 192.938 | 29.04215 | 4.666913 | 0.598484 | ND |
| **6** | 4555.074 | 1015.728 | 191.0349 | 35.57698 | 3.054419 | 1.31105 | 0.658368 |
| **7** | 4576.419 | 1029.428 | 203.6879 | 35.89658 | 1.316404 | 0.584132 | ND |
| **8** | 4461.195 | 955.1268 | 202.1225 | 30.24271 | 5.206774 | 0.668468 | ND |
| **9** | 4568.99 | 1018.525 | 188.4141 | 32.4204 | 4.998017 | 0.691046 | ND |
| **10** | 4547.457 | 912.8226 | 187.3869 | 30.81055 | 4.715448 | 1.988233 | ND |
| **11** | 4561.296 | 848.8992 | 180.7649 | 26.23887 | 3.595204 | ND | ND |
| **12** | 4557.126 | 952.8816 | 201.5665 | 29.71696 | 1.358433 | 0.650937 | ND |
| **13** | 4556.818 | 1013.764 | 184.8285 | 34.17445 | 1.998817 | 0.598423 | ND |
| **14** | 4534.804 | 972.4793 | 192.938 | 29.04215 | 4.666913 | 0.598484 | ND |
| **15** | 4556.031 | 1015.728 | 191.0349 | 35.57698 | 3.054419 | 1.31105 | 0.658368 |
| **16** | 4577.408 | 1029.428 | 203.6879 | 35.89658 | 1.316404 | 0.584132 | ND |
| **17** | 4461.195 | 955.1268 | 202.1225 | 30.24271 | 5.206774 | 0.668468 | ND |
| **18** | 4568.99 | 1018.525 | 188.4141 | 32.4204 | 4.998017 | 0.691046 | ND |
| **19** | 4840.005 | 1044.516 | 247.7369 | 47.76442 | 6.432316 | 2.965568 | ND |
| **20** | 4919.911 | 1062.284 | 238.5912 | 41.41993 | 10.10466 | 2.920007 | ND |
| **21** | 4795.706 | 1026.75 | 231.3039 | 48.21491 | 5.224985 | 2.052282 | 0.720193 |
| **22** | 4936.024 | 1068.127 | 271.1661 | 44.42456 | 13.15157 | 2.375748 | ND |
| **23** | 4969.608 | 1035.26 | 238.2911 | 46.57946 | 6.553656 | 1.225299 | ND |
| **24** | 4941.358 | 1032.786 | 248.5098 | 39.86053 | 7.68568 | 0.703105 | 0.661329 |
| **25** | 4802.459 | 1059.341 | 242.6353 | 50.31834 | 6.571293 | 0.622158 | ND |
| **26** | 5005.072 | 1005.658 | 229.0736 | 54.09691 | 10.05788 | 3.367213 | 2.627077 |
| **27** | 4636.298 | 1058.008 | 265.2057 | 53.47075 | 8.259246 | 1.853537 | 0.638709 |
| **28** | 4736.223 | 979.5359 | 251.4125 | 49.90579 | 5.822382 | 2.856412 | ND |
| **29** | 4766.645 | 978.3876 | 242.1559 | 42.49134 | 6.607192 | 1.35624 | ND |
| **30** | 4888.597 | 974.1176 | 251.6059 | 41.3546 | 7.545757 | 0.603953 | ND |

| **QX200 (wild-type)** | | | | | | | |
| --- | --- | --- | --- | --- | --- | --- | --- |
| **[copies/μL]** | 5^0^ | 5^-1^ | 5^-2^ | 5^-3^ | 5^-4^ | 5^-5^ | 5^-6^ |
| **stdev** | **66.4992** | **15.0422** | **8.86668** | **3.2128** | **1.66591** | **0.2669** | **0.00949** |
| **avg** | **1374.81** | **296.641** | **64.8859** | **10.9384** | **2.03436** | **0.70598** | **0.01105** |
| **1** | 1292.343 | 288.801 | 63.35279 | 11.79025 | 3.367984 | ND | ND |
| **2** | 1264.244 | 288.6342 | 56.22996 | 8.549725 | ND | ND | ND |
| **3** | 1369.001 | 307.2323 | 63.85386 | 10.47977 | ND | ND | ND |
| **4** | 1329.523 | 291.4447 | 63.13098 | 13.52895 | 0.666235 | 0.5984234 | ND |
| **5** | 1360.495 | 274.3809 | 57.87585 | 9.232911 | 0.583263 | 0.5984843 | ND |
| **6** | 1335.177 | 273.9674 | 62.20434 | 12.43971 | 1.221672 | ND | 0.658368 |
| **7** | 1346.959 | 284.2488 | 53.84881 | 3.896501 | 0.658184 | ND | ND |
| **8** | 1274.029 | 283.2148 | 53.56071 | 8.886849 | 0.650721 | ND | ND |
| **9** | 1392.599 | 288.966 | 58.38576 | 6.476936 | 1.249305 | ND | ND |
| **10** | 1298.825 | 291.5085 | 63.35279 | 12.44561 | 3.367984 | ND | ND |
| **11** | 1270.775 | 290.6312 | 56.22996 | 9.119928 | ND | ND | ND |
| **12** | 1374.385 | 309.1305 | 65.21623 | 10.47977 | ND | ND | ND |
| **13** | 1334.882 | 292.6925 | 63.13098 | 13.52895 | 0.666235 | 0.5984234 | ND |
| **14** | 1365.887 | 275.8231 | 57.87585 | 9.232911 | 0.583263 | 0.5984843 | ND |
| **15** | 1339.546 | 274.6151 | 62.20434 | 12.43971 | 1.221672 | ND | 0.658368 |
| **16** | 1355.228 | 285.6455 | 54.46919 | 3.896501 | 0.658184 | ND | ND |
| **17** | 1278.337 | 284.627 | 53.56071 | 8.886849 | 0.650721 | ND | ND |
| **18** | 1400.565 | 289.6813 | 58.38576 | 6.476936 | 1.249305 | ND | ND |
| **19** | 1404.499 | 315.5306 | 64.53503 | 16.66674 | 1.286182 | ND | ND |
| **20** | 1429.683 | 312.5718 | 70.88836 | 11.06992 | 3.60781 | 1.4599127 | ND |
| **21** | 1384.257 | 311.459 | 75.45022 | 13.59361 | 1.306029 | 0.6840542 | ND |
| **22** | 1470.549 | 312.6378 | 69.67997 | 8.640989 | 1.251897 | ND | 0.679589 |
| **23** | 1493.089 | 331.3393 | 67.00438 | 13.61393 | 1.786999 | ND | ND |
| **24** | 1491.446 | 304.8398 | 82.68019 | 12.88128 | 2.36429 | ND | 0.661329 |
| **25** | 1417.207 | 310.5753 | 71.3965 | 14.18574 | 5.376239 | 0.6221585 | ND |
| **26** | 1478.632 | 291.2803 | 78.24198 | 13.50093 | 2.681262 | ND | 0.656714 |
| **27** | 1427.416 | 305.8582 | 86.06991 | 12.18389 | 7.078999 | 0.6178131 | ND |
| **28** | 1449.975 | 302.0718 | 65.61086 | 9.624408 | 4.075365 | ND | ND |
| **29** | 1393.715 | 323.0205 | 66.89342 | 16.41179 | 1.801593 | 0.6781006 | ND |
| **30** | 1421.133 | 302.7976 | 81.25868 | 13.99093 | 3.482056 | 0.6039532 | ND |

| **QIAcuity (mutant)** | | | | | | | |
| --- | --- | --- | --- | --- | --- | --- | --- |
| **[copies/μL]** | 5^0^ | 5^-1^ | 5^-2^ | 5^-3^ | 5^-4^ | 5^-5^ | 5^-6^ |
| **stdev** | **264.927** | **84.1511** | **26.2766** | **14.2279** | **3.43659** | **0.85977** | **0.20171** |
| **avg** | **5415.55** | **1247.97** | **275.706** | **43.3624** | **5.62548** | **1.48375** | **0.06183** |
| **1** | 4865 | 1119 | 249.9 | 43.79 | 9.1 | 2.13 | ND |
| **2** | 5065 | 1131 | 236.2 | 36.39 | 7.24 | ND | ND |
| **3** | 4727 | 1089 | 235.2 | 33.96 | 5.98 | 2.16 | 0.54 |
| **4** | 5328 | 1326 | 305.5 | 58.26 | 11.72 | 3.18 | 0.53 |
| **5** | 5418 | 1323 | 325.3 | 65.84 | 11.72 | 2.2 | ND |
| **6** | 5186 | 1280 | 332.3 | 64.83 | 7.66 | 1.61 | 0.53 |
| **7** | 5652 | 1335 | 315.9 | 67.93 | 9.64 | 0.53 | 1.05 |
| **8** | 5631 | 1331 | 323.7 | 60.19 | 8.33 | 2.74 | ND |
| **9** | 5469 | 1330 | 293.3 | 63.54 | 7.06 | 1.08 | ND |
| **10** | 5391 | 1197 | 299.7 | 48.41 | 10.16 | 2.66 | 0.53 |
| **11** | 5514 | 1205 | 281.1 | 42.55 | 10.59 | 2.2 | ND |
| **12** | 5373 | 1157 | 266.2 | 48.04 | 7.12 | 2.15 | ND |
| **13** | 5440 | 1251 | 275.1 | 46.78 | 6.4 | 2.13 | 1.05 |
| **14** | 5471 | 1165 | 281.3 | 53.65 | 7.26 | 2.2 | ND |
| **15** | 5144 | 1167 | 278.1 | 45.91 | 3.83 | 0.54 | ND |
| **16** | 5580 | 1207 | 261.4 | 33.85 | 6.39 | 0.53 | ND |
| **17** | 5359 | 1194 | 245.8 | 38.68 | 3.35 | 1.1 | 0.54 |
| **18** | 5344 | 1132 | 237.7 | 30.77 | 2.19 | 0.54 | 0.53 |
| **19** | 5312 | 1228 | 268.6 | 22.03 | 2.66 | 0.53 | 0.53 |
| **20** | 5332 | 1188 | 259.5 | 19.88 | 1.67 | ND | ND |
| **21** | 5092 | 1102 | 232.8 | 12.95 | 1.64 | 0.54 | ND |
| **22** | 5490 | 1307 | 280.7 | 60.75 | 9.06 | 1.06 | ND |
| **23** | 5643 | 1319 | 291.8 | 43.19 | 6.12 | 1.65 | 0.54 |
| **24** | 5092 | 1244 | 281.9 | 58.88 | 5.47 | 1.07 | ND |
| **25** | 5672 | 1371 | 284.3 | 44.6 | 3.2 | ND | 0.52 |
| **26** | 5491 | 1373 | 284.2 | 44.32 | 2.23 | 0.55 | ND |
| **27** | 5404 | 1302 | 279.9 | 50.73 | 2.19 | ND | ND |
| **28** | 5792 | 1326 | 276.2 | 30.11 | ND | 0.53 | ND |
| **29** | 5683 | 1293 | 257.5 | 36.6 | 1.11 | ND | ND |
| **30** | 5346 | 1237 | 243 | 33.46 | 1.1 | ND | ND |

| **QIAcuity (wild-type)** | | | | | | | |
| --- | --- | --- | --- | --- | --- | --- | --- |
| **[copies/μL]** | 5^0^ | 5^-1^ | 5^-2^ | 5^-3^ | 5^-4^ | 5^-5^ | 5^-6^ |
| **stdev** | **77.3713** | **31.9745** | **9.61028** | **4.21936** | **1.11586** | **0.59544** | **0.15401** |
| **avg** | **1541.55** | **360.027** | **79.2952** | **12.5927** | **2.34533** | **0.99857** | **0.4816** |
| **1** | 1460 | 303 | 69.41 | 9.6 | 3.21 | 1.6 | 0.017606 |
| **2** | 1496 | 296.6 | 65.97 | 8.81 | 1.11 | 1.64 | ND |
| **3** | 1402 | 296.4 | 70.89 | 15.33 | 3.81 | 2.16 | ND |
| **4** | 1535 | 376 | 85.27 | 17.77 | 3.73 | 1.06 | 0.54 |
| **5** | 1576 | 373.4 | 96.29 | 16.57 | 3.35 | 0.55 | ND |
| **6** | 1424 | 364 | 97.36 | 20.5 | 2.19 | 0.54 | ND |
| **7** | 1649 | 406.5 | 93.03 | 22.43 | 1.61 | 1.07 | 0.53 |
| **8** | 1663 | 396.1 | 84.77 | 13.78 | 3.33 | 0.55 | 0.53 |
| **9** | 1591 | 369.4 | 91.65 | 12.03 | 2.17 | 0.54 | ND |
| **10** | 1600 | 356.5 | 83.72 | 15.04 | 3.74 | 0.53 | ND |
| **11** | 1537 | 330 | 73.5 | 12.7 | 5.01 | ND | ND |
| **12** | 1454 | 336.4 | 74.75 | 12.94 | 2.74 | 1.07 | ND |
| **13** | 1567 | 376 | 72.96 | 8.05 | 2.13 | 1.06 | ND |
| **14** | 1546 | 351.5 | 79.1 | 13.25 | 2.23 | ND | 0.53 |
| **15** | 1448 | 314.4 | 77.39 | 12.41 | 2.74 | 1.61 | ND |
| **16** | 1639 | 355.1 | 73.48 | 9.66 | 2.13 | 0.53 | ND |
| **17** | 1633 | 350.9 | 80.15 | 9.94 | 0.56 | ND | 0.52 |
| **18** | 1514 | 337.7 | 71.77 | 9.71 | 2.19 | ND | ND |
| **19** | 1533 | 333.8 | 72.48 | 5.37 | 0.53 | ND | ND |
| **20** | 1536 | 335.2 | 64.28 | 6.62 | 1.12 | ND | ND |
| **21** | 1418 | 327.9 | 68.55 | 10.25 | ND | 0.54 | ND |
| **22** | 1602 | 369 | 85.21 | 14.49 | 3.73 | 2.12 | ND |
| **23** | 1600 | 393.9 | 97.31 | 11.06 | 2.78 | 1.65 | 0.52 |
| **24** | 1479 | 348.7 | 74.75 | 14.56 | 2.19 | 1.07 | ND |
| **25** | 1609 | 406.4 | 77.78 | 17.18 | 3.2 | ND | 0.53 |
| **26** | 1634 | 391.5 | 94.47 | 15.5 | 2.23 | 0.55 | 0.52 |
| **27** | 1568 | 384.3 | 72.48 | 16.17 | 2.19 | ND | ND |
| **28** | 1584 | 373 | 77.27 | 10.21 | ND | 0.53 | 0.53 |
| **29** | 1461 | 414.1 | 62.96 | 18.84 | 1.11 | ND | 0.53 |
| **30** | 1384 | 382.9 | 79.62 | 11.32 | 1.1 | ND | ND |

| **digiQuark (mutant)** | | | | | | | |
| --- | --- | --- | --- | --- | --- | --- | --- |
| **[copies/μL]** | 5^0^ | 5^-1^ | 5^-2^ | 5^-3^ | 5^-4^ | 5^-5^ | 5^-6^ |
| **stdev** | **103.338** | **19.0659** | **5.12315** | **3.14532** | **1.57451** | **0.75059** | **0.41898** |
| **avg** | **4911.91** | **986.045** | **195.364** | **37.7382** | **7.6359** | **1.57874** | **0.50006** |
| **1** | 4932.884 | 1018.742 | 197.0021 | 38.59686 | 7.371584 | 1.516188 | ND |
| **2** | 5096.05 | 983.0992 | 196.2269 | 39.18932 | 7.287479 | 2.283982 | ND |
| **3** | 4843.688 | 984.1201 | 197.9787 | 38.95662 | 8.73424 | 1.69889 | 1.401584 |
| **4** | 4822.194 | 983.3832 | 205.4511 | 37.73936 | 8.984786 | 2.086751 | 1.401584 |
| **5** | 4921.403 | 978.6301 | 195.7744 | 39.27245 | 7.745555 | 1.885842 | ND |
| **6** | 4898.016 | 981.8419 | 197.5894 | 40.80542 | 8.078404 | 1.235257 | ND |
| **7** | 4920.404 | 980.4688 | 196.7291 | 39.56609 | 8.024601 | 1.515797 | ND |
| **8** | 4926.04 | 985.5989 | 196.8678 | 39.30937 | 7.804095 | 1.397191 | 0.545704 |
| **9** | 4889.704 | 1011.917 | 196.168 | 39.09851 | 7.946333 | 1.848598 | 0.411852 |
| **10** | 4892.881 | 984.2389 | 196.0581 | 39.65938 | 7.857119 | 1.779777 | 0.025512 |
| **11** | 4902.447 | 980.0686 | 182.5947 | 38.3247 | 7.836496 | 1.681337 | 0.075639 |
| **12** | 4913.869 | 975.5574 | 195.8063 | 39.21683 | 8.375024 | 4.789678 | 0.464582 |
| **13** | 4690.949 | 976.3411 | 194.133 | 37.62209 | 8.095795 | 1.243994 | 0.588416 |
| **14** | 4889.252 | 1044.658 | 196.2516 | 38.44471 | 11.44948 | 1.157998 | 0.045842 |
| **15** | 4900.229 | 982.2854 | 193.7953 | 39.82429 | 7.701432 | 1.608758 | 0.560634 |
| **16** | 4943.245 | 979.3763 | 195.1879 | 37.77412 | 7.647144 | 1.488828 | 0.339778 |
| **17** | 5024.002 | 994.3571 | 192.811 | 38.85402 | 7.222753 | 1.369315 | 1.327193 |
| **18** | 4889.211 | 961.626 | 196.9678 | 37.86426 | 7.976997 | 1.444992 | 0.560634 |
| **19** | 4997.855 | 983.0459 | 195.349 | 39.04855 | 7.721112 | 1.285398 | 0.210647 |
| **20** | 4935.438 | 981.0412 | 184.4091 | 39.03299 | 7.700073 | 0.697643 | 0.205926 |
| **21** | 4863.342 | 990.2803 | 196.3136 | 38.56651 | 7.398685 | 2.242161 | 0.560634 |
| **22** | 4946.426 | 990.9232 | 197.3884 | 39.10192 | 7.806145 | 1.800823 | 0.027043 |
| **23** | 4900.888 | 988.6536 | 195.0358 | 39.05272 | 8.016727 | 1.414677 | 0.560634 |
| **24** | 4867.085 | 988.0749 | 197.3394 | 39.31653 | 6.970725 | 1.681901 | 0.560634 |
| **25** | 4649.612 | 998.8809 | 203.2973 | 36.42483 | 7.119108 | 0.866775 | 0.170645 |
| **26** | 5109.045 | 928.3839 | 185.4248 | 28.82377 | 7.406824 | 0.113626 | 0.577472 |
| **27** | 5060.286 | 976.2079 | 185.7103 | 38.09161 | 4.906206 | 1.116898 | 0.030615 |
| **28** | 4910.544 | 993.4704 | 201.7676 | 27.15017 | 1.094113 | 1.638374 | 0.295449 |
| **29** | 4745.355 | 1004.485 | 201.3061 | 30.99332 | 8.179777 | 1.349492 | 1.167109 |
| **30** | 5074.861 | 971.5834 | 194.1911 | 36.42483 | 8.618277 | 1.121267 | 0.38562 |

| **digiQuark (wild-type)** | | | | | | | |
| --- | --- | --- | --- | --- | --- | --- | --- |
| **[copies/μL]** | 5^0^ | 5^-1^ | 5^-2^ | 5^-3^ | 5^-4^ | 5^-5^ | 5^-6^ |
| **stdev** | **43.0337** | **31.4147** | **2.43652** | **0.64787** | **0.65452** | **0.48792** | **0.62854** |
| **avg** | **1406.84** | **287.278** | **56.6351** | **10.9874** | **2.10141** | **0.61757** | **0.36335** |
| **1** | 1468.323 | 282.7699 | 54.42565 | 11.07727 | 2.594988 | 0.849445 | ND |
| **2** | 1403.154 | 281.7174 | 56.06993 | 11.14784 | 2.256046 | 0.453288 | ND |
| **3** | 1408.839 | 285.1153 | 55.95097 | 11.42127 | 2.327566 | 0.312009 | 2.251029 |
| **4** | 1426.813 | 279.5894 | 57.33283 | 10.74732 | 2.803168 | 0.284066 | 0.114604 |
| **5** | 1413.771 | 278.113 | 56.1946 | 11.27601 | 2.37127 | 1.401584 | ND |
| **6** | 1400.985 | 280.756 | 56.6917 | 10.72212 | 3.100474 | 1.401584 | ND |
| **7** | 1397.982 | 280.9852 | 56.2515 | 11.14033 | 2.271286 | 0.343812 | ND |
| **8** | 1525.315 | 280.7983 | 56.05035 | 11.07977 | 2.194596 | 0.617778 | 0.560634 |
| **9** | 1396.958 | 285.1455 | 56.21832 | 11.23079 | 2.129598 | 0.468334 | 0.415417 |
| **10** | 1415.286 | 283.9825 | 56.14889 | 11.03924 | 2.16011 | 0.690202 | 0.025512 |
| **11** | 1398.441 | 285.7226 | 55.98572 | 10.31935 | 2.220336 | 0.679584 | 0.411852 |
| **12** | 1401.517 | 282.1128 | 56.42778 | 11.15588 | 2.270074 | ND | 0.016838 |
| **13** | 1261.64 | 282.0085 | 54.57162 | 11.49765 | 2.227605 | 0.180369 | 0.027783 |
| **14** | 1443.312 | 312.3718 | 55.99068 | 11.48003 | 3.116149 | 2.460145 | 2.361457 |
| **15** | 1388.348 | 290.7341 | 55.16058 | 11.03354 | 2.262944 | 0.411852 | 0.02041 |
| **16** | 1400.042 | 281.3575 | 56.33145 | 11.28674 | 2.140142 | 0.560634 | 0.124804 |
| **17** | 1440.538 | 284.5888 | 57.11084 | 12.05498 | 0.202716 | 0.113963 | 0.560634 |
| **18** | 1399.51 | 291.503 | 56.02864 | 10.90381 | 2.482288 | 0.33073 | 0.010205 |
| **19** | 1421.192 | 286.3304 | 55.90801 | 11.02576 | 2.252739 | 0.379502 | 0.075639 |
| **20** | 1404.301 | 279.931 | 54.57269 | 11.23013 | 2.097337 | 0.829944 | 0.027783 |
| **21** | 1404.564 | 285.0566 | 56.12286 | 11.10383 | 2.237593 | 0.399431 | ND |
| **22** | 1433.734 | 278.9175 | 57.23224 | 11.18826 | 2.385816 | 1.121267 | 0.560634 |
| **23** | 1407.1 | 285.0076 | 55.79161 | 11.2551 | 2.448461 | 0.900412 | 0.205926 |
| **24** | 1389.305 | 282.4875 | 56.10093 | 12.2695 | 1.595175 | 0.560634 | 0.124804 |
| **25** | 1396.919 | 281.9026 | 52.93952 | 10.70513 | 2.415962 | 0.339778 | 0.411852 |
| **26** | 1436.306 | 191.2507 | 55.45523 | 8.621783 | 1.529808 | 0.284827 | 0.016838 |
| **27** | 1399.377 | 291.4246 | 59.66728 | 10.82746 | 1.251709 | 0.124804 | 0.010205 |
| **28** | 1434.479 | 419.6083 | 66.72561 | 10.12441 | 0.285813 | 0.707882 | 0.054826 |
| **29** | 1346.194 | 308.9193 | 61.28918 | 9.951364 | 1.271348 | 0.281785 | 0.124804 |
| **30** | 1340.961 | 298.1245 | 58.3059 | 10.70513 | 2.139243 | 0.419828 | 0.205926 |

**Supplementary Table 4. Raw data of the volume variance of micro-patterns.**

| Top | | Bottom | |  |  |
| --- | --- | --- | --- | --- | --- |
| Horizon(µm) | **Vertical(µm)** | **Horizon(µm)** | **Vertical(µm)** | **Height(µm)** | **Volume(nL)** |
| 227 | 200 | 166.18 | 146.41 | 98.57 | 3.38 |
| 220 | 198 | 161.05 | 144.95 | 99.42 | 3.27 |
| 222 | 200 | 162.52 | 146.41 | 103.38 | 3.47 |
| 220 | 201 | 161.05 | 147.14 | 97.02 | 3.24 |
| 217 | 198 | 158.85 | 144.95 | 101.82 | 3.31 |
| 218 | 198 | 159.59 | 144.95 | 98.57 | 3.22 |
| 223 | 200 | 163.25 | 146.41 | 96.17 | 3.24 |
| 225 | 200 | 164.71 | 146.41 | 97.02 | 3.30 |
| 222 | 199 | 162.52 | 145.68 | 96.17 | 3.21 |
| 227 | 200 | 166.18 | 146.41 | 97.02 | 3.33 |
| 220 | 201 | 161.05 | 147.14 | 99.42 | 3.32 |
| 218 | 200 | 159.59 | 146.41 | 99.42 | 3.28 |
| 221 | 200 | 161.78 | 146.41 | 96.17 | 3.21 |
| 219 | 201 | 160.32 | 147.14 | 100.97 | 3.36 |
| 226 | 201 | 165.44 | 147.14 | 92.21 | 3.17 |
| 226 | 200 | 165.44 | 146.41 | 91.36 | 3.12 |
| 219 | 200 | 160.32 | 146.41 | 91.36 | 3.03 |
| 227 | 204 | 166.18 | 149.34 | 97.02 | 3.40 |
| 221 | 201 | 161.78 | 147.14 | 96.17 | 3.23 |
| 215 | 200 | 157.39 | 146.41 | 94.61 | 3.08 |
| 217 | 197 | 158.85 | 144.21 | 93.76 | 3.03 |
| 222 | 202 | 162.52 | 147.87 | 91.36 | 3.10 |
| 226 | 196 | 165.44 | 143.48 | 93.06 | 3.12 |
| 227 | 194 | 166.18 | 142.02 | 94.61 | 3.15 |
| 229 | 198 | 167.64 | 144.95 | 89.80 | 3.08 |
| 230 | 200 | 168.37 | 146.41 | 90.65 | 3.15 |
| 201 | 202 | 147.14 | 147.87 | 100.27 | 3.08 |
| 218 | 199 | 159.59 | 145.68 | 97.02 | 3.18 |
| 221 | 200 | 161.78 | 146.41 | 100.97 | 3.37 |
| 236 | 198 | 172.76 | 144.95 | 94.61 | 3.34 |
| 232 | 197 | 169.84 | 144.21 | 96.17 | 3.32 |
| 222 | 201 | 162.52 | 147.14 | 97.02 | 3.27 |
| 232 | 197 | 169.84 | 144.21 | 95.46 | 3.30 |
| 205 | 200 | 150.07 | 146.41 | 97.86 | 3.03 |
| 229 | 198 | 167.64 | 144.95 | 94.61 | 3.24 |
| 224 | 201 | 163.98 | 147.14 | 92.21 | 3.14 |
| 221 | 197 | 161.78 | 144.21 | 93.76 | 3.09 |
| 222 | 197 | 162.52 | 144.21 | 95.46 | 3.16 |
| 230 | 202 | 168.37 | 147.87 | 97.86 | 3.44 |
| 225 | 198 | 164.71 | 144.95 | 94.61 | 3.19 |
| 232 | 202 | 169.84 | 147.87 | 96.17 | 3.41 |
| 204 | 201 | 149.34 | 147.14 | 101.82 | 3.16 |
| 218 | 200 | 159.59 | 146.41 | 99.42 | 3.28 |
| 224 | 201 | 163.98 | 147.14 | 99.42 | 3.38 |
| 225 | 199 | 164.71 | 145.68 | 97.02 | 3.28 |
| 231 | 198 | 169.10 | 144.95 | 97.02 | 3.35 |
| 228 | 203 | 166.91 | 148.61 | 99.42 | 3.48 |
| 224 | 206 | 163.98 | 150.80 | 97.86 | 3.41 |
| 214 | 208 | 156.66 | 152.27 | 99.42 | 3.35 |
| 227 | 201 | 166.18 | 147.14 | 98.57 | 3.40 |
| 231 | 201 | 169.10 | 147.14 | 98.57 | 3.46 |
| 218 | 206 | 159.59 | 150.80 | 102.67 | 3.49 |
| 213 | 210 | 155.93 | 153.73 | 100.97 | 3.41 |
| 228 | 194 | 166.91 | 142.02 | 97.86 | 3.27 |
| 224 | 193 | 163.98 | 141.29 | 93.06 | 3.04 |
| 220 | 194 | 161.05 | 142.02 | 95.46 | 3.08 |
| 223 | 196 | 163.25 | 143.48 | 97.02 | 3.21 |
| 222 | 190 | 162.52 | 139.09 | 97.02 | 3.09 |
| 217 | 192 | 158.85 | 140.55 | 96.17 | 3.03 |
| 232 | 197 | 169.84 | 144.21 | 95.46 | 3.30 |
| 232 | 207 | 169.84 | 151.53 | 94.61 | 3.43 |
| 256 | 196 | 187.40 | 143.48 | 92.21 | 3.50 |
| 247 | 205 | 180.82 | 150.07 | 93.76 | 3.59 |
| 234 | 206 | 171.30 | 150.80 | 93.06 | 3.39 |
| 259 | 201 | 189.60 | 147.14 | 89.80 | 3.53 |
| Average | | | | | **3.27** |
| Stdev | | | | | **0.14** |
| RSD(%) | | | | | **4.39%** |

**Supplementary Table 5. Raw data of measurement results using digiQuark for each ratio of EGFR T790M mutation standard sample.**

|  | Mutant/Total* | #1 | #2 | | #3 | #4 | #5 | #6 | #7 | #8 | | #9 | Average | Stdev | RSD(%) |
| --- | --- | --- | --- | --- | --- | --- | --- | --- | --- | --- | --- | --- | --- | --- | --- |
| Mutant  (FAM) | **2%** | 20.4 | 21.0 | | 27.8 | 18.3 | 20.3 | 21.3 | 20.8 | 22.3 | | 24.6 | **21.9** | **2.8** | **12.83%** |
|  | **1%** | 10.9 | 10.9 | | 10.1 | 10.1 | 9.5 | 10.1 | 6.6 | 11.3 | | 12.7 | **10.3** | **1.7** | **16.06%** |
|  | **0.20%** | 2.9 | 2.5 | | 2.0 | 2.8 | 2.8 | 2.5 | 2.5 | 2.8 | | 2.2 | **2.6** | **0.3** | **11.82%** |
|  | **NTC** | 0.3 | 0.3 | | 0.2 | 0.0 | 0.5 | 0.0 | 0.6 | 0.3 | | 0.0 | **0.2** | **0.2** | **-** |
| Wild-type  (Cy5) | **2%** | 846.2 | 830.3 | | 895.7 | 848.0 | 827.8 | 811.7 | 850.6 | 911.2 | | 1045.8 | **874.2** | **71.8** | **8.22%** |
|  | **1%** | 729.0 | 842.3 | | 897.7 | 752.2 | 772.4 | 848.6 | 811.3 | 834.9 | | 979.1 | **829.7** | **76.9** | **9.26%** |
|  | **0.20%** | 941.8 | 802.4 | | 798.8 | 962.7 | 751.7 | 760.5 | 808.9 | 814.8 | | 721.8 | **818.2** | **82.1** | **10.04%** |
|  | **NTC** | 0.2 | 0.5 | | 0.0 | 0.3 | 0.5 | 0.0 | 0.9 | 0.0 | | 0.0 | **0.3** | **0.3** | **-** |
| Ratio  (Mutant/Total) | **2%** | 2.41% | 2.53% | | 3.11% | 2.15% | 2.45% | 2.63% | 2.44% | 2.44% | | 2.35% | **2.50%** | **0.3%** | **10.43%** |
|  | **1%** | 1.50% | 1.30% | | 1.13% | 1.35% | 1.23% | 1.20% | 0.82% | 1.36% | | 1.30% | **1.24%** | **0.2%** | **15.37%** |
|  | **0.20%** | 0.31% | 0.31% | | 0.26% | 0.29% | 0.37% | 0.33% | 0.31% | 0.35% | | 0.30% | **0.32%** | **0.0%** | **10.57%** |
| Linearity (R^2^) | | | | **y=1.2167x+0.0006** | | | | | | | **R^2^=0.9994** | | | | |

*** Standard material, KRISS RM111-10-509 (based on QX200 results)**
